# Double Staining Immunohistochemistry and Digital Pathology: Moving Towards Standardization of the Proliferative Index Evaluation in Meningiomas

**DOI:** 10.3390/jpm16030148

**Published:** 2026-03-04

**Authors:** Viscardo Paolo Fabbri, Giuseppe Broggi, Giovanna Attolini, Vincenzo L’Imperio, Fabio Pagni, Angela Guerriero, Stefano Marletta, Francesco Fiorentino, Stefania Caramaschi, Claudia Malagoli, Albino Eccher, Rosario Caltabiano

**Affiliations:** 1 Pathology Unit, Department of Laboratory Medicine and Anatomical Pathology, AOU Policlinico di Modena, 41125 Modena, Italy; giovanna.attolini@gmail.com (G.A.); malagoli.claudia@aou.mo.it (C.M.); albino.eccher@unimore.it (A.E.); 2 Department of Medical and Surgical Sciences and Advanced Technologies “G.F. Ingrassia”, Anatomic Pathology, University of Catania, 95124 Catania, Italy; giuseppe.broggi@gmail.com (G.B.); rosario.caltabiano@unict.it (R.C.); 3 Department of Medicine and Surgery, Pathology, University of Milano-Bicocca, IRCCS (Scientific Institute for Research, Hospitalisation and Healthcare) Fondazione San Gerardo dei Tintori, 20900 Monza, Italy; vincenzo.limperio@gmail.com (V.L.); fabio.pagni@unimib.it (F.P.); 4 Department of Pathology Unit, Padua University Hospital, 35128 Padua, Italy; angela.guerriero@aopd.veneto.it; 5 Division of Pathology, Humanitas Istituto Clinico Catanese, 95045 Catania, Italy; stefano.marletta92@gmail.com; 6 Pathology Unit, Santa Maria Goretti Hospital of Latina-ASL Latina, 04100 Latina, Italy; frfiorentino@hotmail.it; 7 Section of Pathology, Department of Medical and Surgical Sciences for Children and Adults, University of Modena and Reggio Emilia, 41124 Modena, Italy; stefania.caramaschi@unimore.it; 8 Clinical and Experimental Medicine PhD Program, Department of Biomedical, Metabolic, and Neural Sciences, University of Modena and Reggio Emilia, 41121 Modena, Italy

**Keywords:** meningioma, double staining immunohistochemistry, digital pathology, proliferative index

## Abstract

**Background:** Although Ki-67 is not included among the grading criteria in the current WHO Classification of Tumours of the Central Nervous System (CNS), it provides valuable, albeit limited, prognostic information. Immunohistochemistry for Ki-67 can reveal uneven proliferation patterns and assist in the assessment of mitotic counts. Several studies indicate that meningiomas with a proliferation index > 4% show recurrence rates comparable to CNS WHO grade 2 (atypical) tumors, while tumors with an index > 20% are associated with mortality rates similar to CNS WHO grade 3 (anaplastic) meningiomas. Issues related to Ki-67 assessment include interobserver variability, the use of different cut-off values among pathologists, and the presence of a complex inflammatory tumour microenvironment, which may lead to an overestimation of the proliferative index (PI). **Methods:** In this study, we describe how Double Staining Immunohistochemistry (dIHC) with EMA/Ki-67 better highlights neoplastic meningothelial cells compared with single-stain evaluation. Furthermore, the application of Digital Pathology provides quantitative digital data that allow a more accurate assessment of proliferation. **Results:** Ki-67 expression varied by grade, with digital image analysis (dIHC) showing high agreement with manual assessments. dIHC improved accuracy in evaluating diagnostic and proliferative markers within tumor samples. **Conclusions:** dIHC combined with DP can support and standardize the evaluation of the proliferative index in meningiomas in routine diagnostic practice.

## 1. Introduction

Meningiomas are extra-axial tumours of the central nervous system (CNS), accounting for approximately 36.4% of all CNS neoplasms [1]. This neoplasm exhibits a broad morphological spectrum encompassing 15 subtypes [2]. According to the 2021 WHO Classification, meningiomas are classified as grade 1, grade 2, or grade 3 [2]. The diagnosis of atypical meningioma, the most common type of grade 2 meningioma, is based on major and minor histopathologic criteria. The major criteria include high mitotic activity (>4 mitoses per 10 high-power fields, HPF) and brain invasion. The minor criteria—of which at least three must be present—include high cellularity, small cells with a high nuclear-to-cytoplasmic ratio, prominent nucleoli, sheeting (uninterrupted patternless or sheet-like growth), and foci of spontaneous (non-iatrogenic) necrosis.

In WHO classifications, chordoid and clear cell meningiomas were noted to have a higher recurrence rate (RR) than the average CNS WHO grade 1 meningioma and were therefore assigned to grade 2. Chordoid meningiomas often lack any other high-grade histopathological features, but they have recurrence rates analogous to those of atypical meningiomas; clear cell meningiomas are associated with more aggressive behaviour, including recurrence and occasional cerebrospinal fluid seeding, pending larger studies to confirm the higher rates of recurrence.

However, larger and prospective studies are needed to validate these designations and to identify additional prognostic biomarkers. Similarly, rhabdoid and papillary morphologies qualify for CNS WHO grade 3, irrespective of other features of malignancy.

Recently, molecular data have supported the development of an integrated classification (as in gliomas), incorporating genetic and epigenetic alterations such as SMARCE1 mutations (clear cell subtype), BAP1 loss (rhabdoid and papillary subtypes), KLF4/TRAF7 mutations (secretory subtype), TERT promoter mutations and/or homozygous deletions of CDKN2A/B (grade 3), and H3K27me3 loss of nuclear expression, which is associated with a potentially worse prognosis [2,3].

DNA methylation-based meningioma classification defines clinically more homogeneous groups and demonstrates greater predictive power for tumour recurrence and prognosis than the WHO classification. This approach may help stratify meningioma patients into observation-only versus adjuvant treatment groups [4].

Surgical resection remains the primary treatment for meningiomas of all grades [5]. Adjuvant radiotherapy (aRT) is the standard of care for grade 3 tumours, remains controversial for grade 2, and is generally not indicated for grade 1 lesions.

Based on morphological stratification and treatment, anaplastic meningiomas have a poor prognosis, with median 5-year progression-free survival (PFS) and overall survival (OS) of 13.9 and 56.9 months, respectively [6]. More variability is reported for grade 2 and grade 1 tumours: atypical meningiomas show a 5-year OS of 81% and a RR of 28% [7], whereas grade 1 meningiomas have a RR ranging from 7% to 20%, despite an OS consistently above 90% [8], even when gross total resection (GTR) is achieved.

In the era of personalized medicine (PM), the heterogeneous clinical behaviour of grade 1 and grade 2 meningiomas and their variable therapeutic responses highlight the need for additional prognostic parameters to guide postoperative management, helping to identify patients at low or high risk of progression.

PM is defined as the opportunity to move beyond a “one-size-fits-all” approach to diagnosis, follow-up, and therapy, toward individualized patient care. In neurosurgery, PM has become feasible thanks to advances in imaging analysis, and genomic, proteomic, and epigenetic biomarkers that provide clinically relevant information about individual patients. For example, large-scale genetic analyses have revealed mutations in the NF2 pathway in 50–60% of meningiomas. Other genes, including TRAF7, SMO, AKT1, and KLF4, are often mutated in NF2-wildtype tumours [9]. Clinical trials investigating targeted therapies directed against these altered pathways are currently underway.

However, complex molecular analyses and advanced imaging software are not always accessible and can be costly. In this context, immunohistochemical (IHC) evaluation of Ki-67 has emerged as a promising and cost-effective marker in routine pathology, facilitating personalized treatment strategies and tailored follow-up for patients who may require adjuvant therapy beyond surgery.

The negative prognostic significance of a high Ki-67 proliferative index (PI) has been well documented in most solid tumours [10,11,12]; however, its role in meningiomas, while relevant, is not well established. Different cut-off values have been proposed, with considerable variability in the number of slides examined, the selection of fields, and the interpretation of neoplastic “hotspots.” Additionally, evaluating Ki-67 on a single slide may lead to overestimation of PI, as reactive cells within the tumour microenvironment—such as lymphocytes, macrophages, or proliferating endothelial cells—can be mistaken for meningothelial cells.

To overcome these limitations, standardization of Ki-67 evaluation is needed. Various antibodies can also help identify meningothelial cells, aiding in differential diagnosis between meningiomas and other tumours. These markers may be nuclear, cytoplasmic, or membranous. Among nuclear markers, the Progesterone Receptor (PR) is expressed in 67.5% of grade 1, 66.6% of grade 2, and none of grade 3 tumours [13]. The most useful cytoplasmic markers include Somatostatin Receptor type 2 (SSTR2) [14,15] and CD13 [16], each expressed in up to 90% of cases. Epithelial Membrane Antigen (EMA or MUC1), characterized by membranous positivity, remains the most widely used diagnostic marker for meningiomas [17]. EMA is a well-established marker of epithelial differentiation and apical membrane polarity. Beyond its general membranous expression, it is consistently expressed in meningothelial, glandular, epithelial-derived tissues and is particularly useful for highlighting cell borders and luminal surfaces. In double IHC assays, EMA allows reliable identification of the meningothelial compartment, facilitating accurate spatial colocalization and interpretation of the second marker of interest [18]. The aim of this paper is to improve the reproducibility and standardization of the proliferative index assessment in meningiomas, particularly in grade 1 and grade 2 cases, through the integrated use of Double Staining Immunohistochemistry (dIHC) with EMA/Ki-67 and Digital Pathology.

## 2. Materials and Methods

This study is conducted as a retrospective analysis. This study included 97 non-consecutive meningioma cases operated on between January 2020 and December 2022. All data were handled in accordance with the ethical principles of the Declaration of Helsinki, with full respect for participant privacy and confidentiality. The study is part of a broader project on central nervous system lesions (approved by Area Vasta Emilia Romagna Centro Ethics Committee, protocol number 467-2021-OSS-AUSLBO-21070-ID2239) on 21 April 2021.

Histological features, WHO grade, and Ki-67 (monoclonal antibody clone 30-9) expression were independently evaluated by four pathologists: three reviewers were considered “senior” (each with over 10 years of experience in neuropathology slide evaluation), and one was defined as “junior” (1 year of experience). Ki-67 was routinely assessed by light microscopy, considering the average Ki-67 index in four “hot spots” per slide. Since the specimens were always included and evaluated in their entirety, the most representative tissue block for each case was selected subjectively by the pathologists.

A double-staining immunohistochemistry (dIHC) was performed using EMA (rabbit monoclonal antibody E-29, Ventana Medical Systems–Roche Diagnostics, Cat. No. 05878900001) and Ki-67 (rabbit monoclonal antibody 30-9, Ventana Medical Systems–Roche Diagnostics, Cat. No. 05278384001). The procedure followed the method described by Krenacs et al. [19]. Double immunohistochemistry was performed on formalin-fixed, paraffin-embedded (FFPE) tissue sections using an automated Ventana Roche immunostaining platform (Ventana Medical Systems, Tucson, AZ, USA), according to the manufacturer’s instructions. Four-micrometer–thick sections were deparaffinized and subjected to heat-induced antigen retrieval using Cell Conditioning 1 (CC1, pH 8.5).

CC1 (Ventana Roche) is a reagent solution used in immunohistochemistry (IHC) protocols, specifically for antigen retrieval. It is often part of the process for preparing tissue samples for staining by unmasking or “retrieving” antigens that may have been hidden or altered during tissue fixation. The solution helps to restore the tissue’s antigenic sites, making them more accessible for antibodies during staining. pH 8.5 indicates the pH level of the solution, which is slightly alkaline to help break the cross-links (created by formalin fixation).

Following antigen retrieval, endogenous peroxidase activity was blocked using the Ventana peroxidase inhibitor. The first primary antibody, anti–epithelial membrane antigen (EMA), was applied and incubated under optimized conditions on the automated platform. The primary antibody should be diluted between 1:100 and 1:1000 and incubated at 4 °C overnight or for 1–2 h at room temperature (RT). The secondary antibody should be diluted between 1:500 and 1:2000 and incubated at RT for 30–60 min. For antigen retrieval, use CC1 solution (pH 8.5) and incubate at 95–100 °C for 20–40 min. EMA immunoreactivity was detected using an HRP-based multimer secondary antibody system (ultraView Universal DAB Detection Kit, Ventana), with 3,3′-diaminobenzidine (DAB) as chromogen, resulting in a brown membranous staining pattern.

After completion of the first staining sequence, an additional blocking step was performed to prevent antibody cross-reactivity. The second primary antibody, anti–Ki-67, was then applied and incubated according to the manufacturer’s recommendations. Ki-67 was detected using an alkaline phosphatase-based multimer secondary antibody system (ultraView Universal Red Detection Kit, Ventana), with Fast Red chromogen, yielding a red nuclear signal.

Sections were subsequently counterstained with hematoxylin, blued, and mounted according to standard Ventana protocols. Appropriate positive and negative controls were included in each staining run. The use of distinct antigen retrieval conditions, detection systems, and chromogens allowed clear discrimination between EMA-positive cell membranes (brown) and Ki-67-positive proliferating nuclei (red), enabling accurate assessment of proliferative activity within EMA-expressing cells.

Slides were scanned using a Deepinto slide scanner (Menarini Diagnostics s.r.l. Florence, Italy) and analyzed with the corresponding digital software. The software allowed adjustment of magnification (4×, 10×, 20×, and 40×), zooming and focusing across Z-stacks, measuring, annotation, and image processing, as well as selection of output file formats.

The PI was determined by combining dIHC and Digital Pathology analysis. Yellow circles indicated EMA+/Ki-67+ cells (proliferating meningothelial neoplastic cells), whereas red circles indicated EMA−/Ki-67+ cells (cells belonging to the tumour microenvironment). Digital results were independently reviewed by four pathologists and compared with previously reported Ki-67 values.

To ensure accurate assessment of Ki-67 using digital methods, “hot spot” areas were selected—regions exhibiting the highest density of Ki-67-positive tumor cells—analogous to the approach commonly used in breast cancer evaluation. Specifically, 3–5 fields with the most intense nuclear staining were identified at low magnification, and Ki-67 positivity was then quantified at high magnification (400×) within these fields and per square millimeter (mm^2^). This method allows for a representative measure of tumor proliferative activity [20]. Regions adjacent to necrotic areas were excluded from the analysis, as it is known that necrotic regions and their surroundings often exhibit higher mitotic and proliferative activity, which could bias the assessment of representative tumor proliferation [2].

Notably, it is well established in the literature that EMA staining can be heterogeneous (particularly in higher-grade meningiomas). Also, in our study, some variability in staining intensity was observed; however, only tumor areas showing unequivocal EMA expression were included in the analysis.

## 3. Results

This preliminary study included 97 non-consecutive meningioma cases operated on between January 2022 and December 2022. The cohort comprised 42 men and 55 women, ranging in age from 40 to 82 years (median age 62 years). Tumors were distributed across multiple anatomical sites. Histological grading identified 72 (74%) WHO grade 1, 19 (19%) grade 2, and 6 (7%) grade 3 meningioma. All the clinical and pathological data have been collected in Table 1.

Ki-67 expression showed a clear grade-dependent pattern across the analyzed cases. Grade 1 tumors were characterized by low proliferation indices, with Ki-67 values generally ranging between 4% and 10%. In this subgroup, assessments performed by the expert and the young pathologist were largely comparable, indicating minimal inter-observer variability. Digital image analysis (dIHC) closely mirrored manual evaluations, demonstrating a high level of agreement and supporting the reliability of Ki-67 assessment in low-grade tumors. It is important to note that while we acknowledge the WHO 2021 Classification suggests that tumors with a proliferation index > 4% may have recurrence rates similar to those of Grade 2 meningiomas [2], our study identified Grade 1 tumors with Ki-67 values ranging from 4% to 10%. We believe that tumor heterogeneity and other histopathological factors, such as mitotic activity, lack of necrosis, and absence of anaplasia, may explain why these tumors were classified as Grade 1 despite having a higher proliferation index. Additionally, the variability in Ki-67 values among Grade 1 tumors may reflect the individual biology of the tumor and patient-specific factors.

In Grade 2 tumors, Ki-67 expression was intermediate, with values typically spanning from 12% to 25%. Compared to Grade 1 cases, a higher degree of variability was observed between expert and young pathologist evaluations. While dIHC measurements generally fell within the same range as manual scoring, discrepancies were noted in a subset of cases. These findings suggest that Ki-67 assessment in intermediate-grade tumors is inherently more complex, likely reflecting increased biological heterogeneity and the challenges associated with borderline proliferative indices.

Grade 3 tumors exhibited high Ki-67 expression, frequently exceeding 25% and reaching values of up to 40%. Both observers consistently identified elevated proliferation activity in these high-grade lesions, confirming the robustness of manual assessment in clearly aggressive tumors. Notably, dIHC tended to provide slightly more stable and homogeneous values, particularly in cases with very high Ki-67 expression, suggesting a potential advantage of digital analysis in reducing variability at the upper end of the proliferation spectrum.

**Chart 1:**
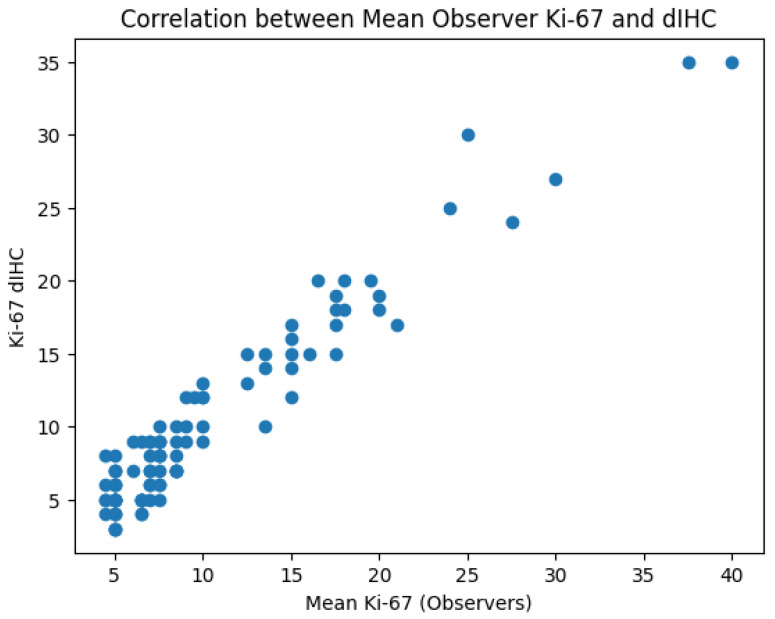
A strong positive correlation (Pearson’s r = 0.96, *p* < 0.001; Spearman’s ρ = 0.89, *p* < 0.001) was observed between the mean Ki-67 values assessed by the observers and those obtained by digital immunohistochemistry, indicating a high level of agreement between manual and digital evaluation across the full range of proliferative activity.

A strong and statistically significant correlation was observed between the mean Ki-67 values assessed by the observers and those obtained by digital immunohistochemistry (Pearson’s r = 0.96, *p* < 0.001; Spearman’s ρ = 0.89, *p* < 0.001), indicating a high level of agreement between manual and digital evaluation (see Chart 1).

**Figure 1:**
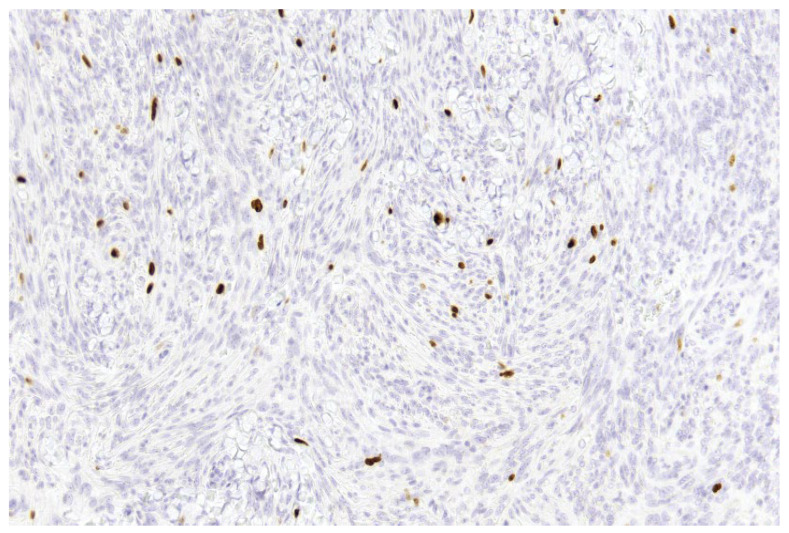
Example of a single-slice Ki-67 staining in a grade 1 meningioma (case 1). Brown-stained nuclei indicate cells in the proliferative phase (10× magnification).

**Figure 2:**
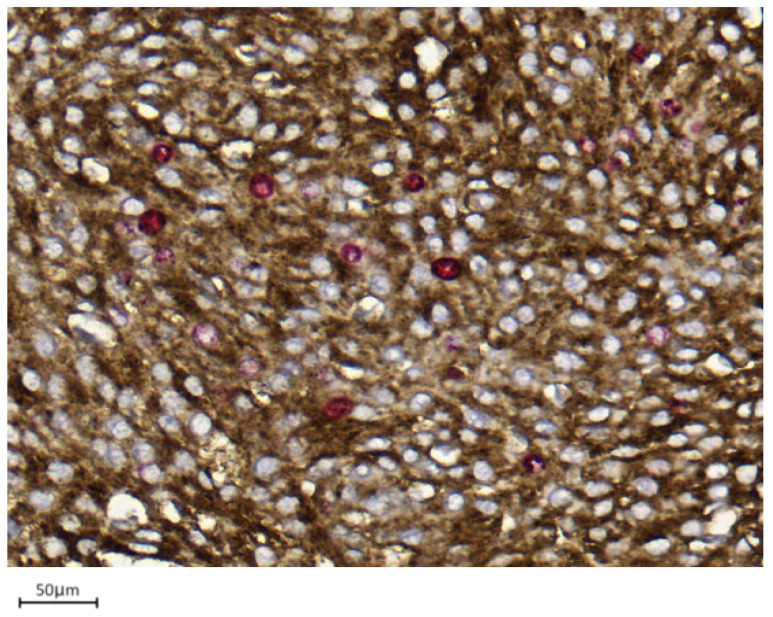
Example of double immunohistochemistry (dIHC) in a grade 1 meningioma (case 7). Meningothelial cells show brown membrane staining, while Ki-67 is visualized in red (40× magnification).

**Figure 3:**
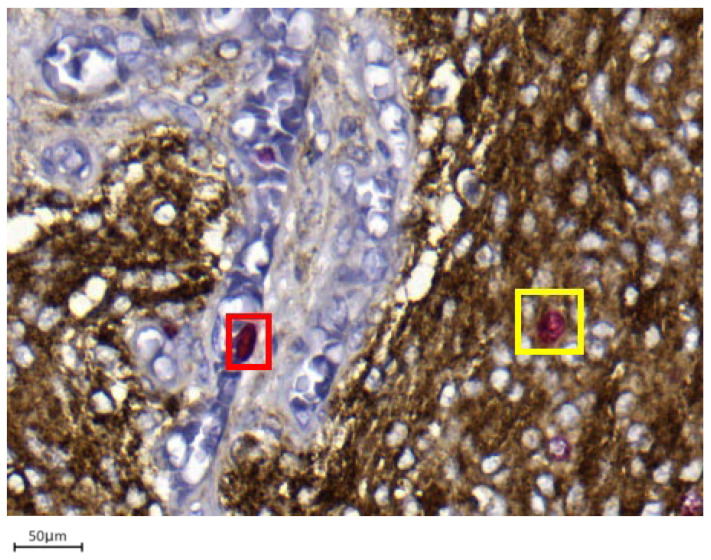
Only EMA/Ki67 in yellow square cells represent proliferating meningothelial cells in a grade 1 meningioma (case 7). Red-only cells in red square may correspond to microenvironmental elements, such as endothelial cells or small lymphocytes (40× magnification).

**Figure 4:**
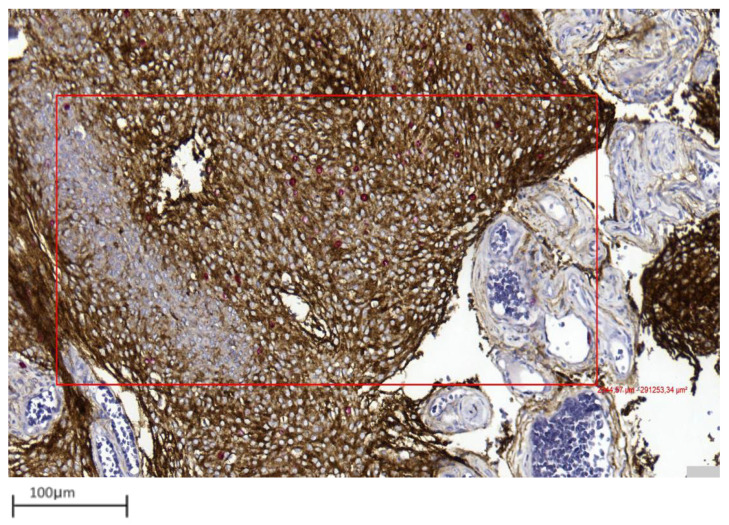
Representative image showing area selection performed with a digital tool in a grade 1 meningioma (case 17) (10× magnification).

**Figure 5:**
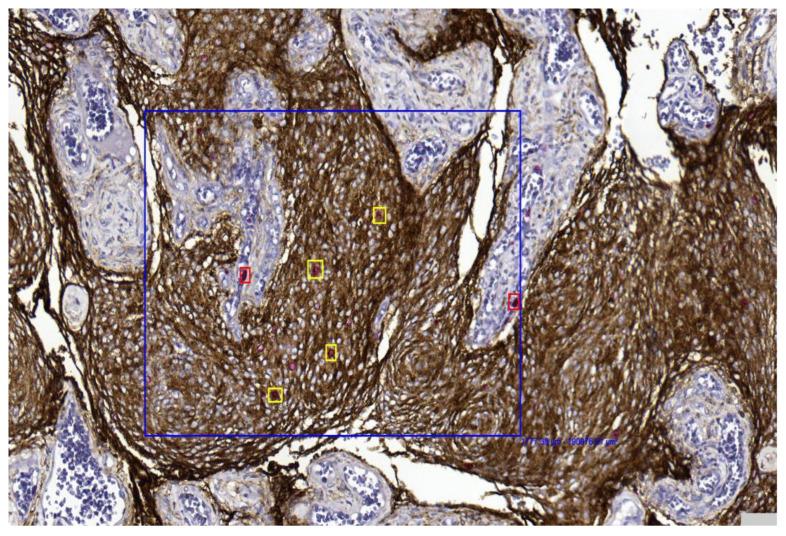
The digital tool selected the area (blue box) and initiated the count of proliferating neoplastic meningothelial cells (yellow) and proliferating microenvironmental elements (red) in a grade 1 meningioma (case 17) (10× magnification).

**Figure 6:**
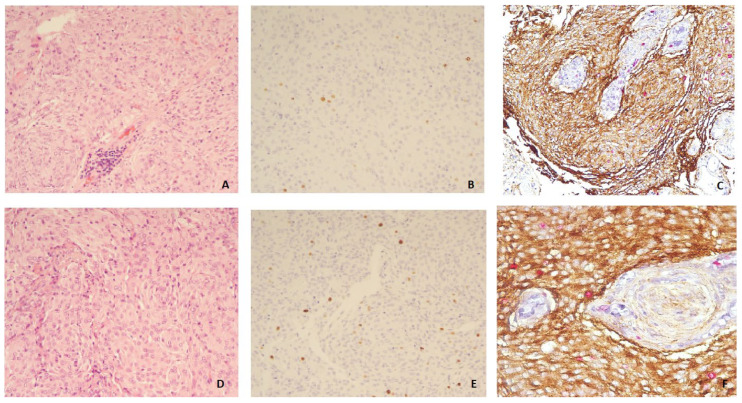
Grade 1 meningioma: H&E (10×) (**A**), Ki-67 on single slide (10×) (**B**), and double immunohistochemistry (dIHC) EMA/Ki-67 (10×) (**C**); Grade 2 meningioma: H&E (10×) (**D**), Ki-67 on single slide (10×) (**E**), and dIHC EMA/Ki-67 (20×) (**F**).

Furthermore, the comparison between the PI assessed by double-staining immunohistochemistry combined with Digital Pathology (dIHC/DP) and the conventional single-slice Ki-67 count gave several important methodological advantages:

- Simultaneous evaluation of diagnostic and prognostic markers: Pathologists can assess, within the same histological section, a diagnostic marker (EMA) and a proliferative marker (Ki-67). This results in a reduction in the time needed to evaluate the Ki-67 index.
- Improved discrimination of neoplastic versus non-neoplastic cells: Unlike single-slice Ki-67 immunostaining (Figure 1), dIHC detects both markers on the same section, allowing precise differentiation between proliferating meningothelial tumour cells and components of the non-neoplastic microenvironment. EMA highlights the cytoplasm of meningothelial cells in brown, while Ki-67 marks proliferating nuclei in red. Only double-positive (EMA+/Ki-67+) cells are included in the PI count (Figure 2), whereas Ki-67-positive but EMA-negative elements (e.g., macrophages, lymphocytes, endothelial cells) are excluded (Figure 3).
- Integration with Digital Pathology: Digital slide scanning enables high-resolution image acquisition, allowing users to measure the selected fields (Figure 4), perform accurate cell counting (Figure 5), and virtually store or share annotated images. Distinct 1 mm^2^ areas were selected, and 100 neoplastic cells were counted in four “hot spots.” Digital magnification and markup tools allowed better discrimination of neoplastic cells from surrounding macrophages, vessels, and lymphocytes, ensuring higher accuracy and reproducibility (Figure 5). Two representative examples illustrating the differences in PI evaluation between single-slice Ki-67 staining and dIHC/DP are presented in Figure 6.

## 4. Discussion

Ki-67 is a well-established immunohistochemical marker of cellular proliferation. High Ki-67 expression correlates with poor clinical outcomes in several cancers. The Ki-67 protein, first identified by Scholzer and Gerdes in the 1980s, is expressed during all active phases of the cell cycle (G1, S, G2, and M) but is absent in quiescent cells (G0) [21]. Nuclear Ki-67 detection by immunohistochemistry (IHC) in formalin-fixed, paraffin-embedded tissue is a reliable and routinely used diagnostic and prognostic tool.

The role of Ki-67 in meningiomas has been explored as a diagnostic, prognostic (for RR and overall survival [OS]), and predictive biomarker. As a prognostic parameter, it may help stratify WHO grade 1 and grade 2 meningiomas into low- and high-risk categories for disease progression, beyond traditional histopathological criteria. Low-risk tumours may require only postoperative surveillance, whereas high-risk cases might benefit from adjuvant therapy.

In daily practice, IHC evaluation of Ki-67 supports mitotic count assessment and correlates well with histological grade. In the study by Telugu et al. [22], the mean proliferative indices (PI) for WHO grade 1, 2, and 3 meningiomas were 3.1%, 7%, and 14.2%, respectively. Several studies indicate that meningiomas with Ki-67 > 4% have recurrence rates comparable to atypical meningiomas, while those with PI > 20% behave similarly to anaplastic tumours [23].

Retrospective studies and meta-analyses have consistently shown that Ki-67 serves as a predictive marker for postoperative recurrence, particularly in low-grade meningiomas. In WHO grade 1 tumours, a Ki-67 index above 6% has been strongly associated with an increased risk of local recurrence following resection. Nowak et al. reported recurrence in 53 of 535 patients (17.7%) at a median follow-up of 31.5 months [24]. In a multivariate analysis by Lee et al. [25], the Ki-67 index was the most powerful predictor of recurrence in atypical meningiomas, outperforming tumour size, Simpson grade, and mitotic activity, with a recurrence rate of 25%.

Moreover, radiomic machine learning classifiers predicting Ki-67 levels ≥ 5% were associated with shorter progression-free survival (PFS) than those with Ki-67 < 5% [26]. A meta-analysis including 43 studies (5012 patients) confirmed that higher Ki-67 expression correlated with worse OS [25], though subgroup analyses showed variability based on ethnicity, tumour grade, and cut-off definitions.

Ki-67 may also act as a predictive biomarker for aRT in resected atypical and anaplastic meningiomas. Five-year PFS rates were 42.3% versus 20.0% for patients with Ki-67 ≤ 10% and >10%, respectively. Similarly, for stereotactic radiosurgery (SRS) delivered at a median dose of 18 Gy, 3-year local control rates were 100%, 74%, and 25% for low (Ki-67 < 5%), intermediate (Ki-67 5–10%), and high (Ki-67 > 10%) PI groups, respectively.

However, existing literature presents several limitations. Variability in defining Ki-67 cut-off values (often arbitrarily set at 4%), the retrospective nature of most studies, inconsistent clinical data (tumour size, grade, surgical extent), and language or publication bias all restrict generalizability. Therefore, well-designed, large-scale prospective studies are necessary to validate these findings.

In routine practice, Ki-67 evaluation is typically performed on a single histological section, which may lead to overestimation of the PI due to the inclusion of proliferating non-neoplastic elements—such as macrophages, lymphocytes, or endothelial cells—that are Ki-67-positive but not meningothelial.

To address these issues, we performed, to our knowledge for the first time, a dIHC combining EMA and Ki-67, followed by Digital Pathology analysis. This approach improves the accuracy of identifying neoplastic meningothelial cells while allowing reproducible digital quantification of proliferative activity. EMA (or MUC1) was selected over other meningioma markers because of its membranous staining pattern, which cannot be confused with the nuclear Ki-67 signal. EMA is a transmembrane glycoprotein expressed in epithelial, meningothelial, and hematopoietic cells, whose overexpression may influence junctional integrity and cell adhesion.

Our study shows a good concordance between expert and young pathologist evaluations, particularly in low- and high-grade tumors. Inter-observer variability increased in Grade 2 cases, where Ki-67 assessment proved more challenging. Digital image analysis showed a high level of agreement with manual scoring and appeared to reduce variability, especially in tumors with intermediate to high proliferative activity. Discrepancies may be attributed to the small cohort size and the limited variability in proliferative activity or microenvironmental composition among the selected cases.

Furthermore, dIHC and DP offer several practical advantages:

- dIHC simultaneously visualizes diagnostic (EMA) and proliferative (Ki-67) markers on a single section, highlighting only the true proliferative meningothelial fraction.
- Digital Pathology facilitates standardization of field selection, enables accurate and shareable digital PI counts, and supports image archiving and telepathology.
- Digital Pathology and dIHC reduce the time required for proliferative index assessment.

Technical efficiency: The use of dIHC reduces laboratory workload, sample handling time, and physical storage space, thereby decreasing overall operational costs [27].

In conclusion, although preliminary and limited by the small sample size, this study proposes a promising and practical approach for improving the reproducibility of Ki-67 proliferative index assessment in meningiomas. Validation in larger cohorts is needed to confirm the potential advantages of dIHC/DP compared with traditional single-slice Ki-67 immunostaining.

Future studies should integrate PI data with clinical, surgical, and radiological parameters, and explore the application of artificial intelligence (AI) to develop objective prognostic and predictive models, particularly to distinguish low-risk from high-risk cases in WHO grade 1 and grade 2 meningiomas.

All these findings could also be associated with data emerging from recent studies, which have highlighted the prognostic value of biomarkers in meningiomas. A recent systematic review and meta-analysis highlighted a range of molecular markers, including Ki-67 and p53, as being associated with recurrence and overall prognosis in meningioma patients [28]. Ki-67, in particular, shows variable distribution across tumor regions, and its quantification provides insights into proliferative activity, which can correlate with tumor grade and potential aggressiveness [29]. Moreover, the combined assessment of p53 and Ki-67 has been proposed as a promising predictor of postoperative recurrence, suggesting that integrating multiple biomarkers may improve prognostic accuracy [29].

## Figures and Tables

**Table 1:** Clinical and pathological data. dIHC = double-staining immunohistochemistry. Age: yo = years-old. Gender: M = male, F = female. Site: 1 = frontal lobe, 2 = temporal lobe, 3 = parietal lobe, 4 = occipital lobe, 5 = spine.

	Age (yo)	Gender	Site	Grade	Ki67-Expert (%)	Ki67-Young (%)	Ki-67 Expert (%)	Ki67-Expert (%)	dIHC (%)
Case 1	82	M	1	1	5	5	5	5	7
Case 2	47	F	1	1	6	7	5	5	7
Case 3	64	M	1	1	5	5	6	7	6
Case 4	62	F	1	1	7	7	5	5	6
Case 5	58	M	1	1	4	5	4	6	5
Case 6	66	F	1	1	8	7	8	9	9
Case 7	71	F	1	1	9	8	7	9	7
Case 8	40	F	3	1	10	8	10	8	10
Case 9	50	M	3	1	5	7	5	5	9
Case 10	62	M	2	1	5	5	6	7	5
Case 11	70	M	5	1	15	15	15	10	12
Case 12	62	F	4	1	10	8	8	10	9
Case 13	50	M	3	1	8	7	8	9	8
Case 14	51	F	2	1	5	8	5	5	5
Case 15	55	M	1	1	5	5	5	5	4
Case 16	49	F	5	1	6	7	5	5	4
Case 17	73	M	4	1	5	5	6	7	5
Case 18	68	M	4	1	7	7	5	5	7
Case 19	59	M	1	1	9	8	10	10	10
Case 20	80	M	1	1	8	10	10	9	12
Case 21	77	F	2	1	10	10	15	15	13
Case 22	65	M	4	1	5	5	5	5	5
Case 23	50	M	1	1	5	5	5	5	3
Case 24	72	F	5	1	5	5	6	7	5
Case 25	61	M	1	1	7	7	5	5	6
Case 26	65	F	2	1	10	5	10	6	8
Case 27	60	M	2	1	8	7	8	9	9
Case 28	55	M	3	1	9	8	7	9	8
Case 29	53	F	5	1	5	5	5	5	5
Case 30	51	M	4	1	6	7	5	5	5
Case 31	49	M	1	1	5	5	6	7	6
Case 32	56	M	1	1	7	7	5	5	7
Case 33	61	F	1	1	10	10	8	8	10
Case 34	66	F	2	1	8	7	8	9	8
Case 35	70	F	3	1	5	5	7	4	5
Case 36	66	F	3	1	5	5	5	5	4
Case 37	55	F	4	1	6	4	5	5	4
Case 38	79	M	3	1	10	10	10	10	9
Case 39	63	M	3	1	10	10	10	10	12
Case 40	62	M	2	1	15	10	15	15	13
Case 41	76	F	1	1	8	7	8	9	10
Case 42	51	F	4	1	5	8	7	5	5
Case 43	48	M	3	1	5	5	5	5	7
Case 44	53	F	2	1	8	7	8	8	9
Case 45	55	M	4	1	5	5	6	5	3
Case 46	57	F	1	1	7	7	8	8	9
Case 47	48	F	1	1	4	5	4	6	6
Case 48	66	M	1	1	8	7	8	9	5
Case 49	54	M	1	1	9	8	7	9	7
Case 50	51	M	1	1	5	5	5	5	8
Case 51	49	F	3	1	10	10	10	10	12
Case 52	50	F	4	1	9	10	9	9	12
Case 53	52	M	4	1	7	7	5	5	9
Case 54	53	F	5	1	4	5	4	6	8
Case 55	67	F	5	1	8	7	8	5	6
Case 56	68	M	2	1	9	8	7	9	7
Case 57	79	M	2	1	5	5	5	5	6
Case 58	70	M	3	1	6	7	5	5	5
Case 59	66	M	4	1	12	15	9	7	10
Case 60	53	M	4	1	7	7	5	5	8
Case 61	52	F	4	1	4	5	4	6	5
Case 62	75	F	2	1	8	7	8	9	6
Case 63	73	F	2	1	9	8	7	9	7
Case 64	67	F	1	1	5	5	5	5	7
Case 65	66	F	1	1	6	7	7	10	9
Case 66	56	F	1	1	5	5	4	4	3
Case 67	77	F	5	1	7	7	5	5	5
Case 68	57	M	2	1	4	5	4	6	4
Case 69	55	M	2	1	8	7	8	9	7
Case 70	78	M	3	1	9	8	7	9	9
Case 71	79	F	1	1	5	5	5	5	5
Case 72	61	F	1	1	6	7	5	5	4
Case 73	55	F	1	2	15	12	12	10	15
Case 74	54	F	1	2	15	10	10	15	15
Case 75	59	M	4	2	15	20	20	18	19
Case 76	60	F	3	2	18	18	25	15	20
Case 77	65	F	2	2	15	15	15	15	14
Case 78	64	F	5	2	12	18	18	20	15
Case 79	63	M	1	2	15	15	20	15	17
Case 80	70	F	1	2	20	20	20	20	19
Case 81	55	F	1	2	18	15	20	20	20
Case 82	74	F	1	2	20	20	15	20	18
Case 83	65	M	2	2	12	15	15	10	14
Case 84	78	F	2	2	25	14	15	25	20
Case 85	64	F	2	2	20	15	20	17	18
Case 86	60	F	4	2	20	22	20	16	17
Case 87	55	F	4	2	17	15	15	15	15
Case 88	56	F	3	2	15	20	16	20	15
Case 89	57	F	2	2	15	15	15	15	16
Case 90	78	F	1	2	15	20	20	15	17
Case 91	78	F	5	2	16	20	15	15	18
Case 92	69	F	1	3	20	28	30	25	25
Case 93	67	M	3	3	25	30	25	25	24
Case 94	71	M	3	3	30	30	30	30	27
Case 95	66	F	2	3	25	25	25	25	30
Case 96	65	F	1	3	35	40	30	35	35
Case 97	63	F	1	3	40	40	30	40	35

## Data Availability

The original contributions presented in this study are included in the article. Further inquiries can be directed to the corresponding author.
